# Mechanical Regulation of Gene Expression in Gut Smooth Muscle Cells

**DOI:** 10.3389/fphys.2017.01000

**Published:** 2017-12-05

**Authors:** Xuan-Zheng Shi

**Affiliations:** Department of Internal Medicine, University of Texas Medical Branch, Galveston, TX, United States

**Keywords:** mechanical stress, motility, visceral sensitivity, abdominal pain, obstruction, COX-2, NGF, MAPKs

## Abstract

Intraluminal contents and their movement along the gastrointestinal tract create shear stress and mechanical stretch on the gut wall. While the shear stress is important in the initiation of immediate physiological responses, the circumferential mechanical stretch, such as that in obstructive bowel disorders, exerts long-lasting impacts on bowel functions by mainly affecting the deeper muscularis externae. Recent studies demonstrate that mechanical stretch alters gene transcription in gut smooth muscle cells (SMC), and the stretch-altered gene expression (mechano-transcription) may play a critical role in pathogenesis of motility dysfunction and abdominal pain in obstruction. Specifically, stretch-induced cyclo-oxygenase-2 and other pro-inflammatory mediators in gut SMC account for impairments of muscle contractility. Mechano-transcription of pain mediators such as nerve growth factor may contribute to visceral hypersensitivity, by sensitizing primary sensory neurons. This review aims to highlight the novel findings of mechano-transcription in the gut, and to discuss the signaling mechanisms and pathophysiological significance of mechano-transcription.

## Introduction

The gastrointestinal (GI) tract, as the body's largest hollow-organ system, is constantly subjected to mechanical stimuli (Shi et al., 2011). On the one hand, gut smooth muscle generates three types of contracting forces, i.e., tone, rhythmic phasic contraction, and giant migrating contraction (Sarna and Shi, 2006). The main functions of these contractile activities are to mechanically digest foods and to move the intraluminal contents along the GI tract at an adequate pace (Murthy, 2006; Sarna and Shi, 2006; Kraichely and Farrugia, 2007). On the other hand, the presence and movement of intraluminal contents (foods, gas, and fluids) create two types of mechanical forces on the gut wall: shear stress and pressure. Shear stress is a transient force generated at the mucosa surface tangential to the GI tract, whereas intraluminal pressure creates a circumferential stretch perpendicular to the gut wall.

As the shear stress is generated at the mucosa surface, it primarily affects mucosa and submucosa (M/SM). The impacts of shear stress on epithelial cells and enterochromaffin cells (EC) have been previously reviewed (Gayer and Basson, 2009; Linan-Rico et al., 2016). In contrast, the deeper muscularis externae (ME) including smooth muscle cells (SMC) and myenteric plexus are mainly subjected to circumferential stretch. This review focuses on the effects of circumferential stretch on the gut wall, especially the novel findings of mechanical stretch-induced gene expression, a process called “mechano-transcription” (MT). We further discuss the signaling mechanisms and pathophysiological significance of MT in the GI tract.

## Mechanical stretch in obstructive bowel disorders

Under the physiological condition, the intraluminal pressure in the intestine is nearly 0 cmH_2_O (Summers, 1999; Silen, 2005). However, if the movement of intraluminal contents is blocked as in obstruction, the pressure in the obstructed segment may increase to 8~10 cmH_2_O, or more than 30 cmH_2_O when peristalsis occurs (Shikata et al., 1983; Silen, 2005). Obstruction also leads to overload of intraluminal contents proximal to the occlusion, leading to lumen distention. Thus, the circumferential mechanical tension on the gut wall (product of pressure and radius) is greatly increased according to Laplace's law (Russell and Welch, 1990).

Many GI conditions are associated with lumen distention, and thus mechanical stretch. These conditions are classified as obstructive bowel disorders (OBD) (Lin et al., 2012a). Lumen distension in OBD may be due to functional or mechanical or obstruction. Functional obstruction results from neuromuscular dysfunction, such as in ileus, intestinal pseudo-obstruction, idiopathic megacolon, and Hirschsprung's disease (Nunez et al., 2009; De Giorgio et al., 2011). Mechanical obstruction may originate extrinsic to the intestine, e.g., adhesions, hernias, or intrinsic to the intestine, e.g., carcinoma and diverticulitis (Russell and Welch, 1990; Summers, 1999; Silen, 2005). Mechanical bowel obstruction (BO) is the most common OBD. The annual aggregate cost for hospital stay in BO is more than $2.7 billion, topping all other GI conditions (Milenkovic et al., 2006).

Earlier studies have documented a series of functional and morphological abnormalities in obstruction. These include motility dysfunction (Summers et al., 1983; Prihoda et al., 1984), visceral hypersensitivity (Huang and Hanani, 2005), muscle hypertrophy (Gabella, 1975, 1990), and injuries in the enteric nervous system (ENS) and interstitial cells of Cajal (ICC) (Chang et al., 2001; Wedel et al., 2002). If left untreated, these changes may eventually lead to intestinal failure (Thompson, 2006). Even if obstruction is surgically resolved, many patients continue to have long-term bowel dysfunction (Grosfeld and Rescorla, 1993; Langer, 2004; Kim et al., 2005; Menezes et al., 2006).

To address pathophysiology of OBD, early studies focused on pathological changes weeks after the introduction of obstruction (Gabella, 1975; Chang et al., 2001; Bertoni et al., 2004; Won et al., 2006). This approach might have missed important early molecular events, which could eventually lead to the latent functional and morphological changes. Over the last few years, we and others have tested a new theory in the understanding of bowel dysfunction in OBD. We proposed that mechanical stretch regulates expression of “stretch-sensitive” genes in the gut, and that mechano-transcription plays a critical role in bowel dysfunction in OBD.

## Mechanical regulation of gene expression in the gut

To examine mechanical stretch-induced gene expression in the gut, *in vitro* and *in vivo* stretch models have been developed. The Flexcell system is a well-established *in vitro* model to study mechanical stretch in cultured cells (Gayer and Basson, 2009; Shi et al., 2011; Li et al., 2012a). In this system, the computer-regulated bioreactor applies finely controlled multi-axial static or cyclic strains through vacuum pressure to cells cultured on flexible membrane plates. Applying this *in vitro* model of mechanical stretch in the primary culture of rat colon SMC, Lin et al. found that static stretch induced mRNA and protein expression of IL-8, IL-6, MCP-1, iNOS, cyclo-oxygenase-2 (COX-2), but not TNF-α and IL-1β (Lin et al., 2014a). Wehner et al. also used this system and found that static stretch significantly induced iNOS and COX-2 mRNA in intestinal SMC (Wehner et al., 2010).

Mechanical BO is the prototype of OBD. We have used the model of partial colon obstruction to investigate *in vivo* mechanical regulation of gene expression (Shi et al., 2011). To induce BO, a 3-mm wide medical grade silicon band is placed around the mid colon. The size of the band is determined to be 1–2 mm longer than the outer circumference of the colon when the colon is filled with a fecal pellet, allowing a partial obstruction. As intestinal manipulation may be associated with up-regulation of pro-inflammatory gene expression in the gut (Kalff et al., 2000), one has to implement strict sham controls in the model (Shi et al., 2011). We treated the sham control similarly as in obstruction animals with the obstruction band being placed, but released 2 min later. In the obstruction animals, both the distended oral segment and the non-distended aboral segment are taken for comparisons (Shi et al., 2011). If there is any surgery-associated inflammation, it will be detected in the sham and aboral segment. These approaches make it possible to study the specific effect of mechanical stretch *in vivo*. Moreover, the gene expression profiles in the models of obstruction and surgical manipulation-induced inflammation are distinctively different (Shi et al., 2011).

With the *in vivo* model of partial colon obstruction, we screened for “stretch-sensitive” genes in an Affymetrix cDNA array with 28,700 candidate genes included (Shi et al., 2011; Lin et al., 2017b). The transcription of 309 genes was increased more than 2-fold, whereas that of 282 genes was decreased more than 2-fold in the mechanically stretched ME tissues, comparing to the non-stretch controls. Overall, we identified several major groups of genes whose expression is altered by mechanical stretch, including those encoding certain inflammatory mediators (i.e., COX-2), growth factors, neurotrophins, adhesion molecules, extracellular matrix proteins, and some cell signaling proteins.

**Figure 1:**
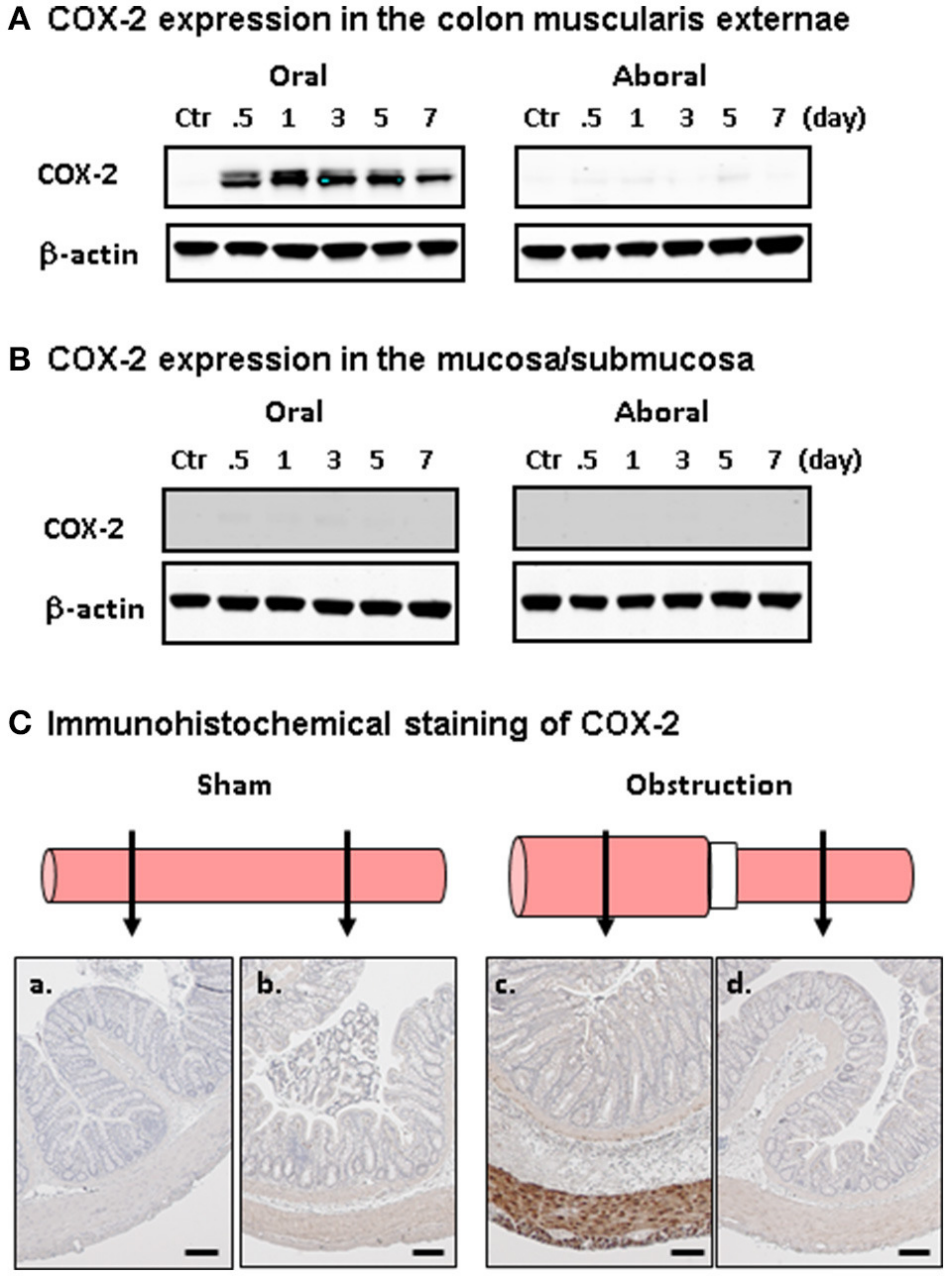
Bowel obstruction induced mechano-transcription of COX-2 selectively in the SMC**. (A)** Western blot detection of COX-2 in the colonic muscularis externa in the oral (left) and aboral (right) segments. **(B)** Western blot detection of COX-2 in the mucosa/submucosa in the oral (left) and aboral (right) segments. **(C)** Immunohistochemical staining of COX-2 expression in the oral **(a,c)** and aboral **(b,d)** colon segments in sham control **(a,b)** and rat with obstruction **(c,d)** for 3 days. Note that COX-2 (stained in brown) is detected only in SMC in distended oral segment. The results were representative of four independent experiments. Calibration bars represent 50 μm. Figure is adapted with permission from Shi et al. (2011) (PMCID: PMC3025501).

Focusing on MT of COX-2 in BO, we further determined if mechanical stretch induces gene expression selectively in the SMC. The levels of COX-2 mRNA and protein in the muscularis externa were dramatically increased in the stretched colon segment oral to obstruction, but not in the non-stretch aboral segment (Shi et al., 2011). We found that COX-2 expression was induced only in the muscle layer, but not in the mucosa or submucosa. Further immunohistochemical studies showed that the increased COX-2 expression occurs selectively in the SMC, but not in the mucosa, submucosa, or myenteric plexus (Figure 1). Interestingly, Choudhury et al. found that MT of COX-2 in colonic SMC was blocked by de-polymerization of actin filaments, or by siRNA silence of smooth muscle specific α-actin (Acta2) (Choudhury et al., 2015). These results indicate that SMC specific α-actin is critical in MT of COX-2 in the colon.

Lin et al. studied the outcomes of lumen distension in different parts of the GI tract by placing the obstruction band in the lower esophagus, pyloric sphincter, ileum, and colon, respectively. They found that expression of COX-2 mRNA and protein was up-regulated dramatically in all the sites where distention was induced (Lin et al., 2012a). Moreover, when lumen distention of the colon was induced with a balloon at a pressure of 40 mmHg for 40 min, the distended segment demonstrated a significant up-regulation of COX-2 in the smooth muscle, not in the mucosa/submucosa layer. However, no induction of COX-2 was detected when the pressure was at 20 mmHg or less, or when the distention period was shorter than 20 min (Lin et al., 2015).

These studies suggest that mechano-transcription of COX-2 is a force- and time-dependent, and smooth muscle-specific phenomenon. Moreover, mechano-transcription is a potent mechanism commonly utilized throughout the GI tract.

## Intracellular signaling mechanisms of mechano-transcription in the gut

Investigations into the signaling mechanisms of mechano-transcription in the gut have begun. To mimic stretch in obstruction, Li et al. applied static mechanical stretch to primary culture of rat colonic SMC (RCSMC) (Li et al., 2012a). Stretch at 18% elongation robustly increased the expression of COX-2 mRNA and protein. Stretch also induced marked phosphorylation of MAPKs including extracellular signal-regulated kinases (ERKs), MAPK p38, and c-Jun N-terminal kinases (JNKs). Treatment of the cells with inhibitors against ERKs, p38, or JNKs inhibited induction of COX-2, suggesting that all three major MAPK members are involved in the regulation of mechano-transcription of COX-2 in RCSMC (Li et al., 2012a).

Mechanical signals must be sensed at the cell membrane level before it is transduced into the cytoplasm and nucleus (Ruwhof and van der Laarse, 2000; Adam et al., 2004; Kanefsky et al., 2006; Lehoux et al., 2006). We found that integrins and stretch-activated ion channels (SACs) (Hu and Sachs, 1997; Gillespie and Walker, 2001; Katsumi et al., 2004) are the main mechanosensors involved in mechano-transcription of COX-2 in RCSMC (Li et al., 2012a). Mechano-transcription of COX-2 is almost completely inhibited by echistatin, a specific inhibitor to ανβ3, the major type of integrins in gut SMC (Kuemmerle, 2006). Echistatin blocked stretch-induced phosphorylation of p38, but not ERKs and JNKs. Inhibition of SAC with either gadolinium (Suchyna et al., 2000) or GsMTx-4 (Ducret et al., 2010) attenuated mechano-transcription of COX-2, and inhibited stretch-activated ERK1, ERK2, p38, and JNKs (Li et al., 2012a).

Subsequent studies found that stretch also activated protein kinase C (PKC) and protein kinase D (PKD) in colonic SMC (Li et al., 2012b). Inhibition of PKCbeta or PKCzeta did not significantly block stretch-induced expression of COX-2. However, PKCdelta inhibitor rottlerin almost completely blocked mechano-transcription of COX-2. PKD inhibitor CID755673 or treatment with PKD siRNA also inhibited mechano-transcription of COX-2. Rottlerin treatment inhibited stretch-induced activation of all ERKs, p38, and JNKs, whereas CID755673 blocked activation of p38, but not ERKs or JNKs (Li et al., 2012b).

**Figure 2:**
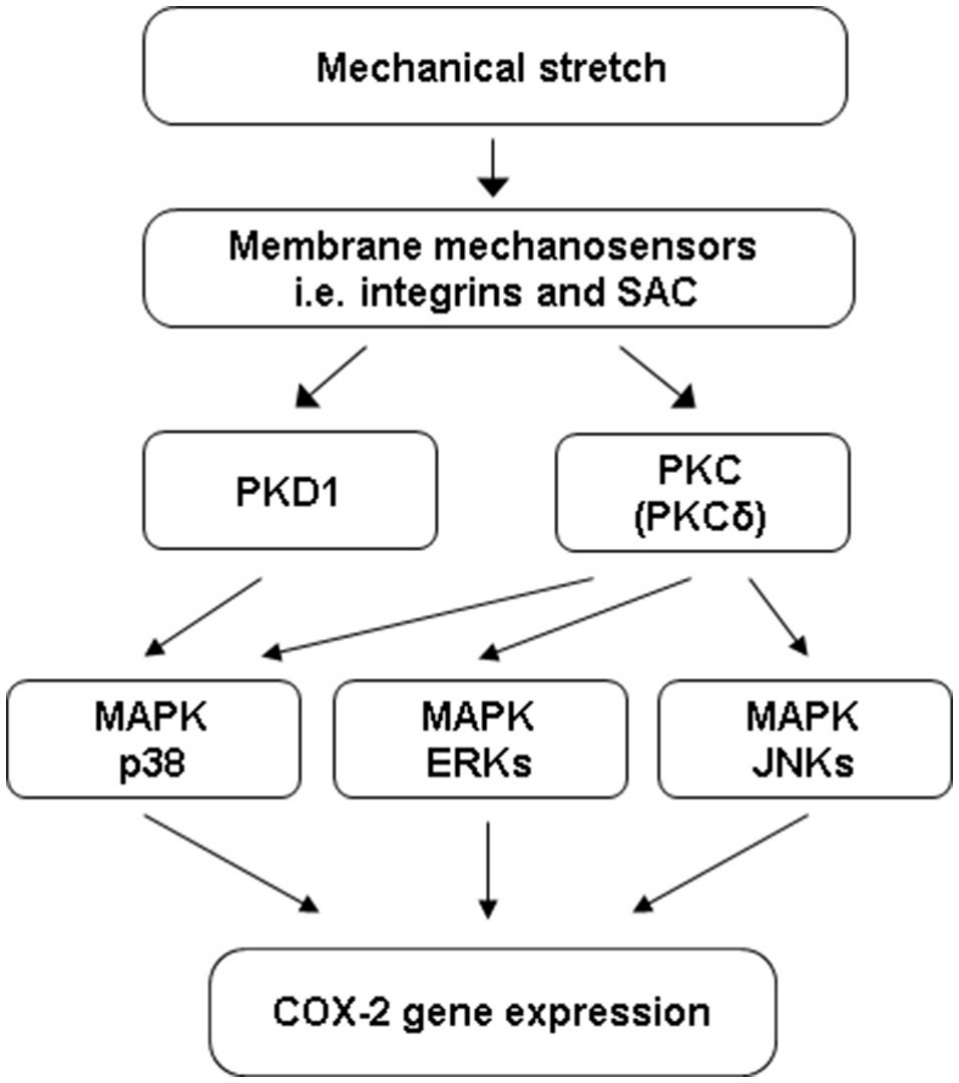
Cell signaling mechanisms of mechano-transcription of COX-2 in colon SMC. Mechanical stretch on gut SMC is sensed by integrins and SAC (stretch-activated ion channels) at the cell membrane level. The mechano-sensors transduce mechanical stimulus to intracellular signaling pathways involving PKC (PKCdelta), PKD (PKD1), and MAPKs to induce mechano-transcription of COX-2 in colonic SMC. PKCdelta is coupled to MAPKs ERKs, p38, and JNKs, whereas PKD1 is coupled to MAPK p38.

Taken together, stretch induced COX-2 in RCSMC is a specific process of transduction of a mechanical stimulus. The mechanosensors integrins and SAC in cell membrane transduce mechanical stimulus to intracellular signaling pathways involving PKCs, PKD, and MAPKs to induce mechano-transcription of COX-2. Current data suggest that PKCdelta is coupled to MAPKs ERKs, p38, and JNKs, whereas PKD is coupled to MAPK p38 (Figure 2).

## Pathophysiological role of mechano-transcription in motility dysfunction in OBD

Previous *in vivo* studies found that gut motility increased in the distended oral segment immediately after induction of obstruction (Prihoda et al., 1984; Summers, 1999). However, motor activity gradually decreased within hours after initiation of obstruction (Fraser et al., 1980; Summers, 1999; Bertoni et al., 2004; Won et al., 2006). The early phase of hyper-motility oral to the obstruction may result from neuronal mechanism similar to the peristalsis reflex that intraluminal accumulation stimulates mechano-receptors in the gut wall to initiate neuronal regulations of ascending excitation and descending inhibition (Prihoda et al., 1984; Grider, 1989). While the initial hyper-motility in the obstructed bowel is a physiological adaptation, the sustained long-term suppression of motility is the most troublesome to patients with OBD. The mechanisms for the sustained hypo-motility are not well understood.

As COX-2 derived prostaglandins (PG) have profound impacts on gut functions (Krause and DuBois, 2000; Fornai et al., 2005), we tested a hypothesis that mechano-transcription of COX-2 play a crucial role in the sustained motility dysfunction in obstruction. Our studies showed that gut smooth muscle contractility was suppressed dramatically in obstruction starting 24 h after the initiation of obstruction in both rats and mice (Shi et al., 2011; Lin et al., 2012a). However, in the COX-2 deficient mice, BO associated suppression of muscle contractility was largely attenuated (Shi et al., 2011), suggesting that mechano-transcription of COX-2 plays a critical role in the suppression of muscle contractility in BO.

Lin et al. tested the *in vivo* effect of COX-2 inhibitor NS-398 in BO. They found that administration of NS-398 either before operation or 3 days after induction of obstruction blocked increase of PGE_2_ and improved colon transit and muscle contractility in the obstructed rats at day 7 (Lin et al., 2012b). These data suggest that COX-2 inhibitor has prophylactic and therapeutic benefits for motility dysfunction in obstruction. They further identified that PGE_2_ and its receptors EP2 and EP4 are involved in the motility dysfunction in obstruction (Lin et al., 2012b).

Mechano-transcription of COX-2 depends on p38 activation in colonic SMC (Li et al., 2012a). Li et al. found that p38 inhibitor SB203580 significantly attenuated induction of COX-2 and improved muscle contractility in obstruction. Thus, inhibition of mechano-transcription pathway may have therapeutic potential for motility dysfunction in OBD.

Although MT of COX-2 may be the most prominent pathway involved in motility dysfunction in BO, other pro-inflammatory mediators induced in obstruction may also play a role. It is discovered that mechanical stretch or obstruction induced expression of IL-6, MCP-1, iNOS, and other pro-inflammatory mediators in gut SMC. These molecules are known to affect motility function in the gut (Lin et al., 2014a). Moreover, medium collected from stretched muscle strips further induced activation of transcription factor NF-κB and expression of pro-inflammatory genes. Thus, mechanical stretch functions as a pro-inflammatory stimulus in the gut, and stretch-induced mediators may exert profound impacts on gut function.

## Pathophysiological role of mechano-transcription in abdominal pain in OBD

Besides motility dysfunction, abdominal pain is another major complaint in BO (Russell and Welch, 1990; Summers, 1999; Silen, 2005), especially among those with inoperable or malignant obstruction (Ripamonti et al., 2001; Jatoi et al., 2004; Ripamonti and Mercadante, 2004). Among patients with advanced malignant obstruction, 92% have distention-associated abdominal pain (Baines et al., 1985). Current analgesic management for BO-associated pain relies on high doses of opioids (Ripamonti and Mercadante, 2004; Roeland and von Gunten, 2009). However, opioids are notoriously known to cause further bowel dysfunction, i.e., constipation and narcotic bowel syndrome (Grunkemeier et al., 2007; Ketwaroo et al., 2013). In addition, abdominal pain is a major symptom in functional obstruction, i.e., chronic intestinal pseudo-obstruction and idiopathic mega-colon (Hanauer and Wald, 2007; De Giorgio et al., 2011). The mechanisms of distension-associated abdominal pain in OBD remain not well understood.

Visceral hypersensitivity is a well-recognized mechanism for abdominal pain (Bielefeldt, 2006; Grundy et al., 2006; Azpiroz et al., 2007; Zhou et al., 2012). It is found that visceral sensitivity is markedly increased in chronic BO. Huang and Hanani reported that the firing threshold of primary afferent neurons was decreased, and that sensory threshold to abdominal stimulation was reduced in colon obstruction mice (Huang and Hanani, 2005). Our study in the rat model of BO found that the dorsal root ganglia (DRG) neurons projecting to the distended colon showed abnormal hyper-excitability with decreased resting membrane potential and rheobase, and increased number of action potentials (Lin et al., 2017b). We tested whether mechano-transcription of pain mediators in the gut contribute to visceral hypersensitivity in BO (Lin et al., 2017a,b).

Neurotrophins such as NGF and BDNF are well-recognized pain mediators (Pezet and McMahon, 2006). Lin et al. found that the expression of NGF mRNA and protein was increased in colonic SMC (not in the mucosa and submucosa layer) in the distended colon oral to obstruction, but not in the non-distended aboral segment, suggesting a mechano-transcription phenomenon (Lin et al., 2017b). Mechanical stretch *in vitro* also led to up-regulation of NGF in RCSMC. Treatment with anti-NGF antibody attenuated sensory neuron hyper-excitability and referred hypersensitivity in BO rats. Furthermore, obstruction led to significant increase of tetrodotoxin-resistant (TTX-r) Na^+^ currents and up-regulation of mRNA expression of TTX-r Na_v_1.8, but not TTX-sensitive Na_v_1.6 and Na_v_1.7 in colon-projecting sensory neurons. These changes were abolished by anti-NGF treatment (Lin et al., 2017b). Thus, mechano-transcription of NGF in colon SMC may play a critical role in visceral hypersensitivity in BO, by acting on TTX-r Na^+^ channels in sensory neurons. Recent study found that mechanical stretch also induces up-regulation of BDNF in SMC, and BDNF may contribute to sensory neuron hyper-excitability in BO by suppressing A-type K^+^ currents (Lin et al., 2017a).

It is yet to determine what other mechanisms may contribute to visceral hypersensitivity in OBD. Although stretch may evoke immediate response in primary afferent nerves (Lin et al., 2017b), it is not known whether sustained distention in obstruction affects nerve endings to release mediators to contribute to visceral sensitivity. Nevertheless, morphological changes in DRG have been described in BO. Williams et al. reported that the size of DRG neurons innervating obstructed intestine was increased (Williams et al., 1993). Huang et al. observed an increased coupling among satellite glia cells in DRG in bowel obstruction (Huang and Hanani, 2005). What caused these changes is not known. It is yet to examine whether the changes in DRG or even second order neurons may contribute to visceral hypersensitivity in obstruction.

## Conclusions and future developments

Recent studies have offered ample evidence that mechanical stretch in obstructive conditions alters gene expression in the gut. Mechano-transcription of COX-2 and other pro-inflammatory mediators in gut SMC plays a critical role in the impairments of muscle contractility in obstruction, whereas stretch-induced pain mediators such as NGF may contribute to visceral hypersensitivity.

Mechanical stretch in obstruction may exert complicate impacts among different cell types in the gut. It is found that obstruction leads to mechano-transcription, hyperplasia and hypertrophy in gut SMC (Gabella, 1990; Lin et al., 2013), whereas it causes injury and disruption in enteric neurons and ICC (Chang et al., 2001; Wedel et al., 2002; Wu et al., 2013; Lin et al., 2014b). The mechanisms underlying these effects deserve further investigation, as injuries of enteric nerves and ICC may contribute to motility dysfunction in OBD. Whether mechanical stretch affects afferent nerve endings in obstruction remains to be determined. If so, this may contribute to visceral sensitivity in OBD. Investigation into the signaling mechanisms of mechano-transcription in the gut has begun (Li et al., 2012a). Further studies may help to develop therapeutic targets to block mechano-transcription pathway for the management of bowel dysfunction in OBD.

## Author contributions

The author confirms being the sole contributor of this work and approved it for publication.

### Conflict of interest statement

The author declares that the research was conducted in the absence of any commercial or financial relationships that could be construed as a potential conflict of interest.
